# Posttreatment with Protectin DX ameliorates bleomycin-induced pulmonary fibrosis and lung dysfunction in mice

**DOI:** 10.1038/srep46754

**Published:** 2017-05-03

**Authors:** Hui Li, Yu Hao, Huawei Zhang, Weiyang Ying, Dan Li, Yahe Ge, Binyu Ying, Bihuan Cheng, Qingquan Lian, Shengwei Jin

**Affiliations:** 1 Department of Anesthesia and Critical Care, Second Affiliated Hospital of Wenzhou Medical University, Zhejiang 325027, China

## Abstract

Protectin DX (10S,17S-dihydroxydocosa-4Z,7Z,11E,13Z,15E,19Z-hexaenoic acid) (PDX), generated from Ω-3 fatty docosahexaenoic acids, is believed to exert anti-inflammatory and proresolution bioactions. To date, few studies have been performed regarding its effect on pulmonary fibrosis. Herein we show that PDX exerts a potential therapeutic effect which is distinct from its anti-inflammation and pro-resolution activity on mice with pulmonary fibrosis. In the present study, we showed that bleomycin (BLM) increased inflammatory infiltration, collagen deposition, and lung dysfunction on day7 after challenged in mice. Posttreatment with PDX ameliorated BLM-induced inflammatory responses, extracellular matrix (ECM) deposition and the level of cytokines related to fibrosis as evaluated by histology analysis, transformation electron microscope (TEM), lung hydroxyproline content and cytokines test. Moreover, PDX improved lung respiratory function, remedied BLM-induced hypoxemia and prolonged life span. In addition, we found that PDX reversed epithelial–mesenchymal transition (EMT) phenotypic transformation *in vivo* and *in vitro*, reinforcing a potential mechanism of promoting fibrosis resolution. In summary, our findings showed that posttreatment with PDX could ameliorate BLM-induced pulmonary fibrosis and lung dysfunction in mice and PDX may be considered as a promising therapeutic approached to fibrotic lung diseases.

Lung fibrosis is the final, common pathological outcome of many chronic inflammatory lung diseases. It is characterized by the excessive accumulation of extracellular matrix (ECM) (such as collagen and fibronectin) in and around inflamed or damaged tissue, which can lead to permanent scarring, progressive dyspnea, pulmonary dysfunction (i.e. reduced lung volumes, impaired gas exchange), and death from respiratory failure^1^. Although fibrogenesis is becoming increasingly recognized as a major cause of morbidity and mortality in most chronic lung diseases, there are few—if any—treatment strategies available that specifically reverse fibrosis^2^.

Specialized pro-resolving mediator (SPM) is a series of endogenic mediators which play a pivotal role during the phase of inflammation resolution. Specific members of each family display potent anti-inflammatory, immune-regulatory and pro-resolving actions *in vivo* and *in vitro* in models of inflammatory diseases^3^. Protectin DX (10(S),17(S)-Dihydroxy-docosa-4Z,7Z,11E,13Z,15E,19Z-hexaenoic acid) (PDX), is produced via sequential lipoxygenation of docosahexaenoic acid (22:6, n-3; DHA) and can be found in inflammatory exudates of a murine model of self-limited resolution^4^. Recent studies have found that PDX inhibited both ROS production and cyclooxygenase activities in human neutrophils. PDX also inhibited blood platelet aggregation through inhibition of COX-1 at submicromolar concentration. In addition, PDX antagonized the thromboxane A2-induced aggregation^5^,^6^. However, the effect of PDX on fibrosis resolution remains unknown.

In the present study, we investigated the effect of posttreatment with PDX on bleomycin(BLM)-induced pulmonary fibrosis in mice. In addition, we observed whether PDX could improve lung function and prolong the survival time. Finally, to better understand the mechanisms of action of PDX, we investigated the effect of PDX on TGF-β1-induced EMT phenotype transformation *in vitro*.

## Results

### BLM-induced pulmonary tissue fibrosis and lung dysfunction in mice

**Figure 1:**
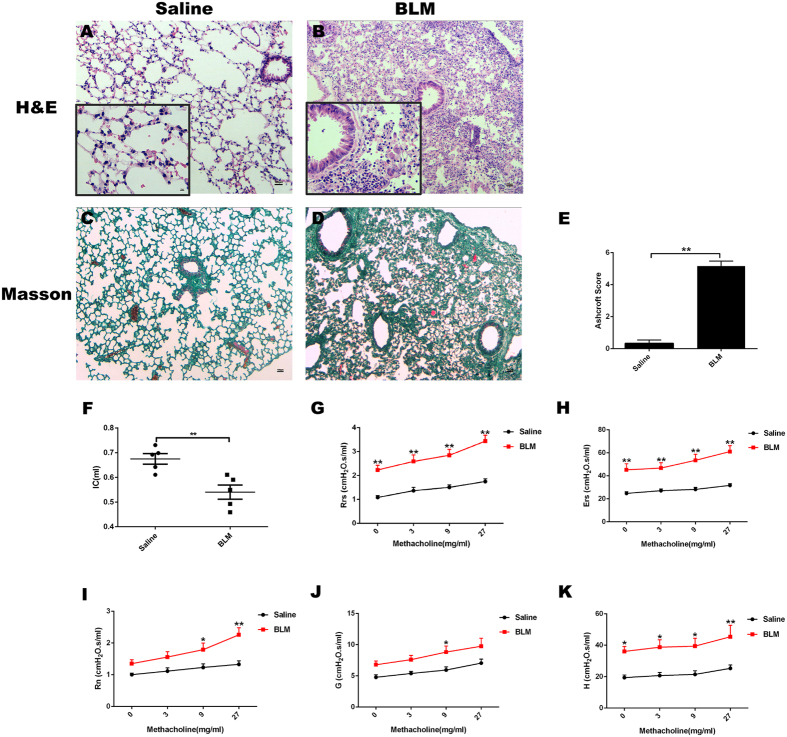
BLM induced pulmonary fibrosis in mice. Mice were treated with saline or bleomycin (BLM) intratracheally (i.t.) at 2.0 mg/kg on day 0. Lungs were removed on day 7 after BLM challenge. Histology of sections with haematoxylin–eosin (HE) for evaluating injured lungs in Saline (**A**) (outside the inset(100x), inset(400x)), and BLM (**B**) are shown. Masson Trichrome staining that detects collagen fibers (MT) (100x) of mice intubated with saline (**C**) or BLM (**D**) are shown. Ashcroft Scores (**E**) indicated the degree of fibrosis. Lung function was determined by the flexiVent system as described in Materials and Methods on day 7 after BLM administration. Inspiratory Capacity (IC) (**F**), respiratory system resistance (Rrs) (**G**), respiratory system elastance (Ers) (**H**), Newtonian resistance(Rn) (**I**), G(tissue damping) (**J**), and H(tissue elastance) (**K**) were shown. Data are presented as mean ± SEM. *P < 0.05,**P < *0.01*. n = 5.

First, we performed an experiment to determine the effect of BLM on the lungs on day 7 after BLM administration by histopathologic analysis and lung function test (Fig. 1). BLM caused a marked inflammatory infiltration (panel B) and collagenous fiber deposition (panels D,E). Compared to saline group, IC in BLM group was decreased significantly, Rrs and Rn, and parameters on behalf of the rigidity of lung tissue including Ers, G, and H upon the methacholine were all increased significantly (panels F–K).

### Posttreatment with PDX reversed BLM-induced lung fibrosis

**Figure 2:**
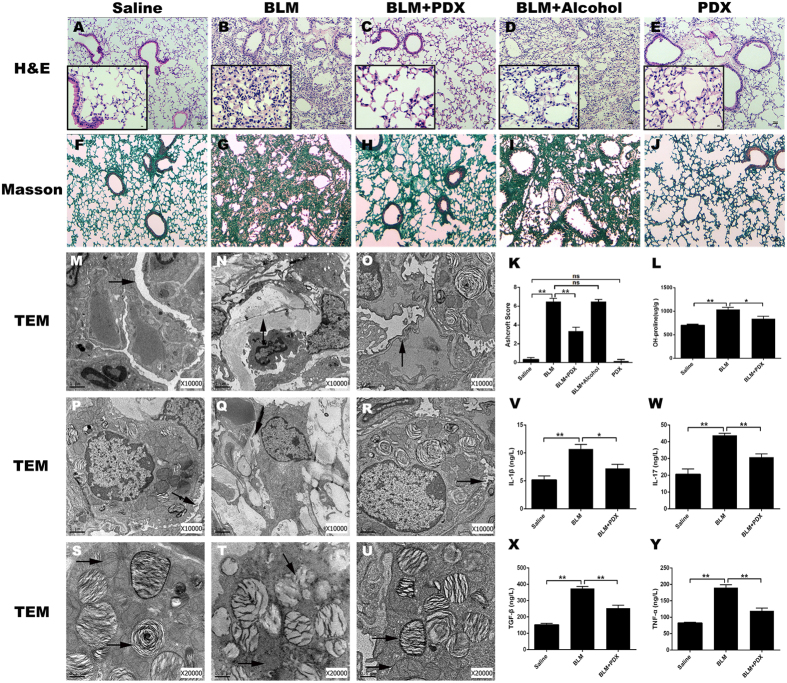
Posttreatment with PDX attenuated BLM induced pulmonary fibrosis. Mice were treated with saline or bleomycin (BLM) intratracheally (i.t.) at 2.0 mg/kg on day 0. From day 8, posttreatment with Protectin DX (PDX) (1 μg/mouse) intraperitoneally (i.p.) followed by boosted 100 ng/mouse every two days. Lungs were removed on day 21 after BLM challenge. Representative lung tissue sections stained with hematoxylin-eosin (HE) at a magnification of 100x (outside the inset) and 400x (inset) (**A–E**). Lung tissue sections stained with Masson’s trichrome to detect collagen content (100x) (**F–J**). Ashcroft fibrosis scores (**K**) indicated the degree of fibrosis. Hydroxyproline content in lungs (**L**). M-U showed PDX ameliorated BLM-induced ultra-structural changes and collage deposition. As arrows shown, PBS group: the alveolar space was clean and integrity (**M**), microvilli (**P**), lamellar bodies with concentric circles or parallel arrangement and mitochondria (**S**) were well observed. BLM group: The alveolar structure severely disrupted, view of ATII cells were decreased (**N**), filled with collagen fibrils (**Q**), increased numbers of evacuation and cacuolation and fusion into macrovesicles were observed (**T**). BLM+ PDX group: the basement membrane integrity, the structure of ATII cells was well-preserved (**O**), the microvilli were clearly visible(**R**), and the lamellar bodies and mitochondrial were all not swollen (**U**). ELISA analysis for IL-1β(**V**), IL-17(**W**), TNF-α(**X**), TGF-β(**Y**). Data are presented as mean ± SEM. ns: not significant, *P < 0.05, **P < *0.01.* n = 5. Alcohol is resolvent.

Representative photomicrographs of slides stained with H&E (panels A–E) and Masson (panels F–J) on day 21 were depicted in Fig. 2. Compared with BLM group, PDX reduced infiltration of inflammatory cell, destruction of lung structure, and deposition of collagen fibers, as evidenced by a similar change in Ashcroft’s fibrosis scoring (panel K). Alcohol had no effect on BLM-induced fibrosis in mice (*P* > 0.05). There was no significant differences between the saline and PDX group (*P* > 0.05). Lung tissue hydroxyproline concentration (panel L) was raised significantly in the BLM group compared with the control group (*P* < 0.01), but greatly attenuated in the PDX treatment group compared with the BLM group (*P* < 0.05).

Next, we sought to explore the effects of PDX treatment on these ultra-structural changes and organelle injuries (Fig. 2M–U). The saline group had a normal pulmonary TEM micrograph. As arrows shown, the alveolar space was patent and clean (panel M). Short microvilli attached to the free surface of ATII (panel P). Lamellar bodies with concentric circles or parallel arrangements and mitochondria were observed in the cytoplasm of ATII cells (panel S). BLM induced marked interstitial fibrin deposition (panel N), lamellar body swelling or vacuolation, and microvilli flattening or disappearance (panels Q,T), whereas PDX posttreatment significantly attenuated BLM-induced ultra-structural changes (panels O,R,U).

### PDX modified BLM induced the production of cytokines related to fibrosis

Subsequently we evaluated if PDX could also modulate the profile of cytokines production induced by BLM challenge (Fig. 2V–Y). As observed, BLM administration enhanced the levels of IL- 1β, IL-17, TNF-α and TGF-β in the lungs compared with the saline group, whereas PDX-treated group presented a significant reduction in these cytokines compared with mice that received BLM alone.

### PDX posttreatment restored lung function after BLM challenge

**Figure 3:**
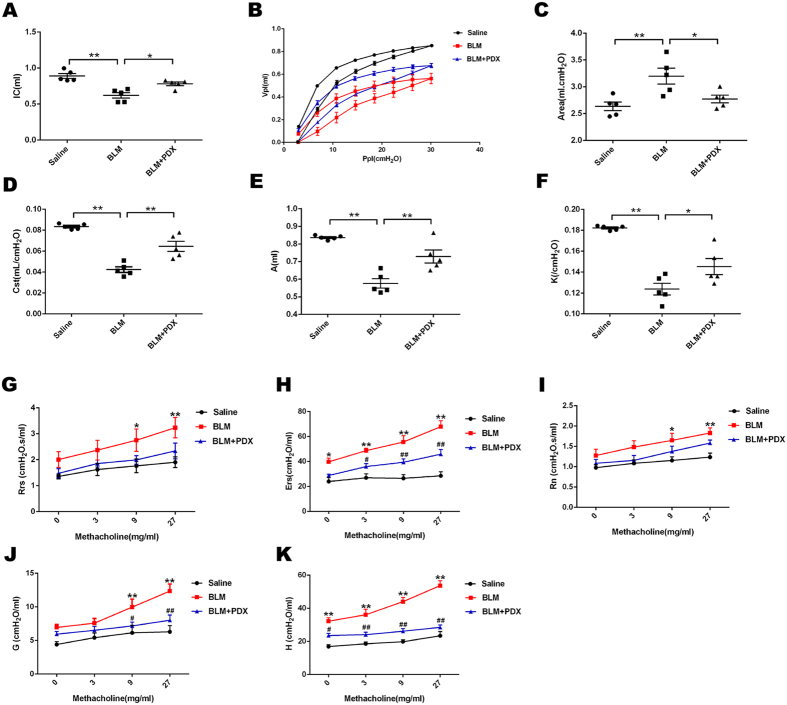
PDX posttreatment restored lung function after BLM challenge. Mice were treated with saline or bleomycin (BLM) intratracheally (i.t.) at 2.0 mg/kg on day 0. From day 8, posttreatment with Protectin DX (PDX) (1 μg/mouse) intraperitoneally (i.p.) followed by boosted 100 ng/mouse every two days. Lung function was determined by the flexiVent system on day 21. Inspiratory Capacity (IC) (**A**), Pressure–volume loop curves (**B**), Area(hysteresis, area in PV loop) (**C**), static compliance (Cst) (**D**), A(total lung capacity) (**E**), K(form of deflating PV loop) (**F**), respiratory system resistance (Rrs) (**G**), respiratory system elastance (Ers) (**H**), Newtonian resistance(Rn) (**I**), G(tissue damping) (**J**), and H(tissue elastance) (**K**) were shown. Data are presented as mean ± SEM. ^*/#^*P* < *0.05*, ^**/##^*P* < *0.01*. *vs saline group, ^#^vs BLM group. n = 5.

To specifically quantify the contribution of the total respiratory system and lung parenchyma in the observed BLM-induced pulmonary phenotype, diverse mechanical properties of the respiratory system were measured (Fig. 3).

A statistically significant reduction of IC was obtained in fibrosis mice when compared with the saline group (*P* < 0.01), whereas in mice treatment with PDX, a statistical increase was observed (*P* < 0.05) (panel A).

PV-loop curve of BLM group was marked shifted down compared with the saline group (panel B), as well as an increased hysteresis (panel C). In addition, Cst, A, K were decreased significantly in BLM mice. Posttreatment with PDX reversed these changes statistically to some extent (panels D–F).

Panels G–K demonstrated that, in BLM-induced group, Rrs, Ers, Rn, G and H were enhanced significantly upon methacholine challenge, whereas PDX improved these parameters to a quite normal level.

### Posttreatment with PDX improved blood-gas exchange after BLM administration

**Figure 4:**
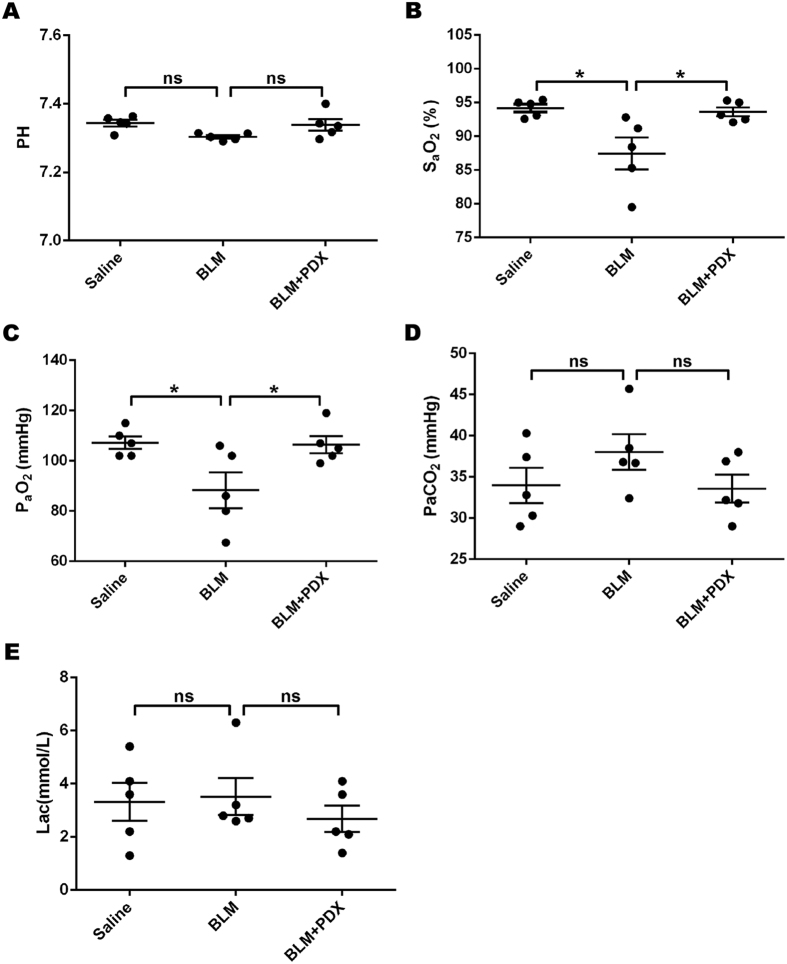
Posttreatment with PDX improved blood gas exchange after BLM administration. Mice were treated with saline or bleomycin (BLM) intratracheally (i.t.) at 2.0 mg/kg on day 0. From day 8, posttreatment with Protectin DX (PDX) (1 μg/mouse) intraperitoneally (i.p.) followed by boosted 100 ng/mouse every two days. Arterial blood-gas analysis was prepared for mice on day 21 after BLM administration. The parameters include PH(**A**), SaO_2_(**B**), PaO_2_(**C**), PaCO_2_(**D**), Lac(**E**). Data are presented as mean ± SEM. ns: not significant, *P < *0.05.* n = 5.

Compared with saline group, the PaO_2_ and SaO_2_ of arterial blood gas were decreased in BLM group (*P* < 0.01), whereas PaCO_2_ had a rising trend without statistically significant (*P* > 0.05). PH and Lac had little change among three groups. By PDX posttreatment, PaO_2_ and SaO_2_ were increased (Fig. 4).

### PDX ameliorated the body weight loss and improved mice survival rate after BLM instillation

**Figure 5:**
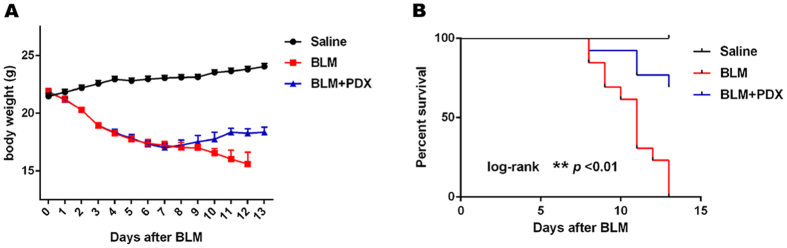
PDX ameliorated the body weight loss and improved mice survival rate after BLM instillation. Mice were treated with saline or bleomycin (BLM) intratracheally (i.t.) at 3.0 mg/kg on day 0. From day 8, posttreatment with Protectin DX (PDX) (1 μg/mouse) intraperitoneally (i.p.) followed by boosted 100 ng/mouse every two days. n = 13 for each experimental group. (**A**) All the body weight of mice in survival analysis was shown. (**B**) Survival curve of the mice was shown. Data was expressed as percentage of mice alive at each time point. Log-rank (Mantel-Cox) test, **p < *0.01*. Data are presented as mean ± SEM.

To assess the protective effects of PDX treatment, mice were injected with BLM at a dose of 3.0 mg/kg in order to induce a strong lung injury and a significant mortality among mice. To get a better understanding of this process, we recorded the changes of body weight daily among the three groups (Fig. 5A). Survival rate analysis was used to evaluate the effect of PDX (Fig. 5B). The survival rate was promoted by PDX in a dose-dependent manner with a concentration of 1μg/mouse producing a maximal effect (see Supplementary Fig. S1). As observed, posttreatment with PDX ameliorated the body weight loss and the death of BLM-treated mice (*P* < 0.01).

### PDX repressed BLM induced EMT *in vivo*

**Figure 6:**
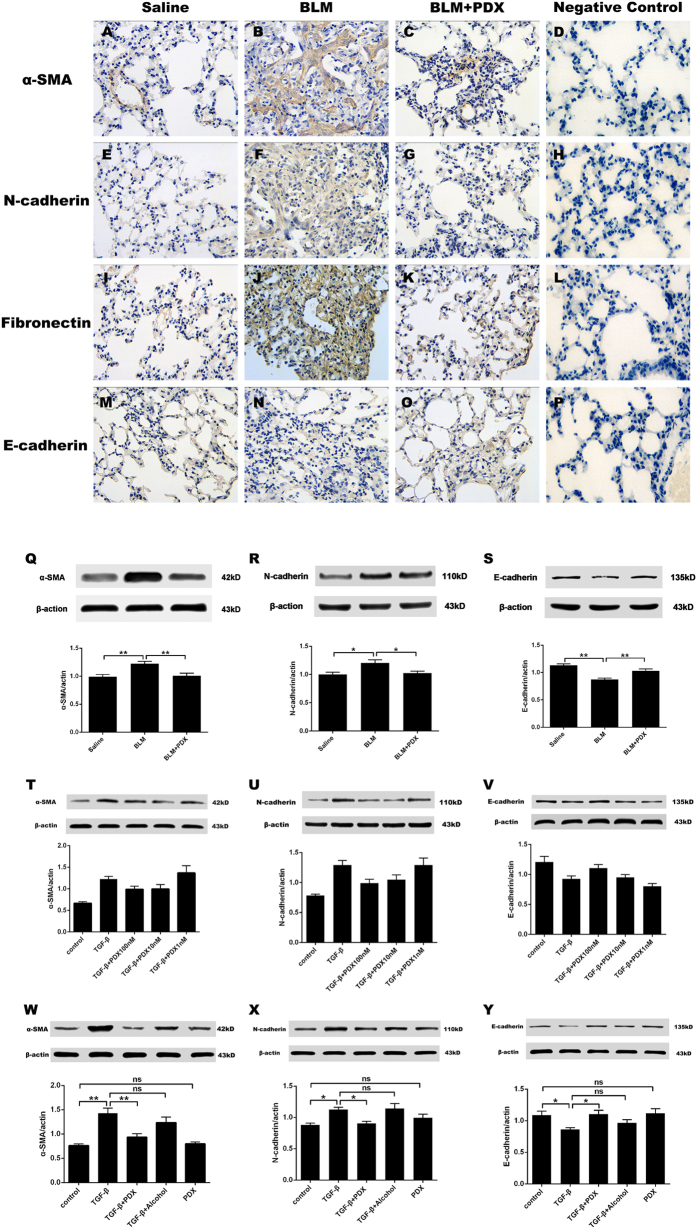
PDX repressed EMT *in vivo* and *in vitro*. Mice were treated with saline or bleomycin (BLM) intratracheally (i.t.) at 2.0 mg/kg on day 0. From day 8, posttreatment with Protectin DX (PDX) (1 μg/mouse) intraperitoneally (i.p.) followed by boosted 100 ng/mouse every two days. Lungs were removed on day 21 after BLM challenge to measure the protein expression of EMT markers by immunohistochemistry (x400) (brown cells represented positive cells) (**A**–**P**) and Western blot (**Q**–**S**). Primary rats alveolar type II epithelial (ATII) cells were administrated with TGF-β1 (10 nM) followed by different concentration of PDX (100 nM, 10 nM, 1 nM) for 48 h to detect the expression of EMT markers including α-SMA, N-cadherin, and E-cadherin protein by western blot (T-Y). Data are presented as mean ± SEM. ns: not significant,*P < 0.05,**P < *0.01.* n = 5. Alcohol is resolvent.

To clarify whether PDX affects BLM induced EMT phenotype, the protein was isolated from lungs and then measured by immunohistochemistry and western blot. Immunohistochemical analysis was used to determine the distribution of α-SMA, fibronectin N-cadherin and E-cadherin in mice lungs after drug intervention (Fig. 6). Positive immunostained cells appeared brown. The number of positive cells expressing α-SMA, fibronectin and N-cadherin was intensified significantly and E-cadherin protein expression was reduced in the BLM group, whereas α-SMA, fibronectin and N-cadherin were decreased and E-cadherin was increased in the BLM+ PDX group compared with the BLM group (panels A–P). As evidenced by a similar change in western blot (panels Q–S).

### PDX inhibited TGF-β1 induced EMT in a dose-dependent manner *in vitro*

Finally, we performed a dose-response experiment comparing the effects of 100, 10 and 1 nM of PDX on TGF-β1-induced EMT phenotypes transformation as assessed (Fig. 6T–V). The dynamic expression of E-cadherin and N-cadherin induced by TGF-β1 initially at 48h with a concentration of 10ng/ml (see Supplementary Fig. S2 and Table S1). As observed, TGF-β1 increased α-SMA, N-cadherin, protein expression and decreased E-cadherin protein expression significantly. Posttreatment with PDX decreased the protein level of N-cadherin, α-SMA and increased the protein level of E-cadherin in primary rats ATII cells following TGF-β1 treated in a dose-dependent manner. However, there was no significant difference between the control and PDX groups (*p* > 0.05) (Fig. 6W–Y).

## Discussion

BLM-induced mice fibrosis is a well-established animal model used in the study pathogenesis of lung fibrosis and the estimation of potential anti-fibrotic agents. The repair procedure is composed of two apparent stages: an enhanced and then weakened inflammatory phase and a fibrotic phase with a low-grade but persistent inflammatory trigger^1^,^7^,^8^,^9^. Plenty of compounds have been shown to inhibit the inflammation and prevent the fibrotic progression in this model which have been suggested be capable for clinical use^10^,^11^,^12^,^13^. However, although at the beginning it is effective, the following failure to control the fibrotic process, when it is initiated, could lead to tissue destruction and dysfunction^14^,^15^,^16^. Compared to prevent the happening of fibrosis, posttreatment strategy is more essential to patients with fibrosis, so as to improve the life span. In the present study, we identified lung tissues fibrosis and lung dysfunction on day 7 after BLM challenge, which illustrates fibrosis has been initially formed in lungs. We were interested in the potential effects of PDX in the fibrotic phase.

**Figure 7:**
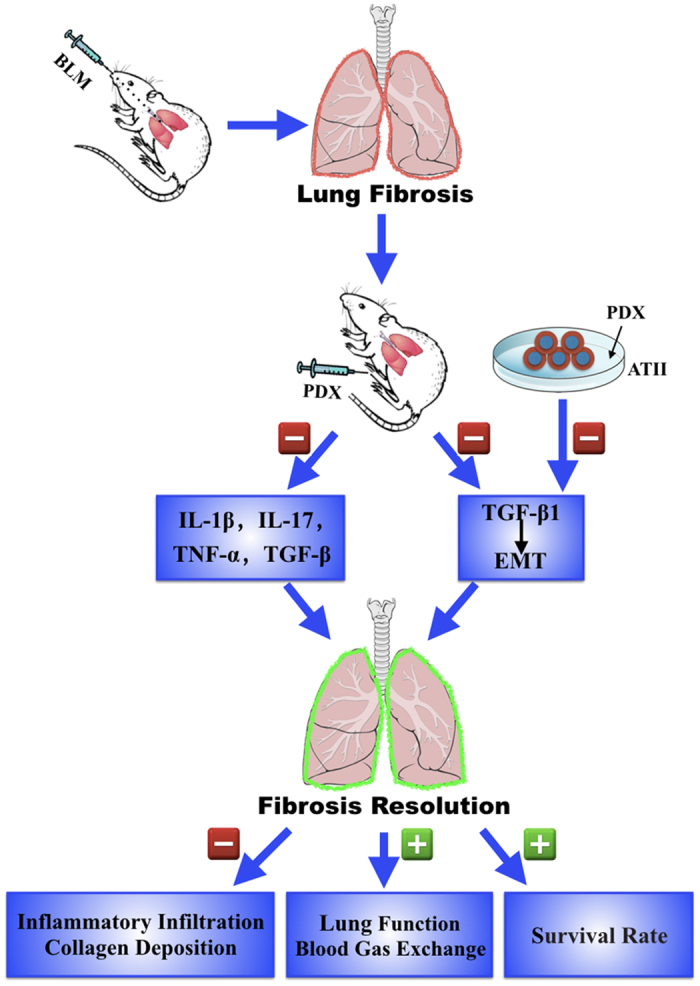
Effects of PDX on lung fibrosis *in vivo* and *in vitro*.

In this work, we provided evidence of ameliorating fibrotic effect by PDX posttreatment in BLM-induced pulmonary fibrosis, promoting the resolution of fibrosis. PDX suppressed inflammatory infiltration and deposition of ECM, reduced expression of pro-fibrotic cytokines, inhibited EMT phenotype, consequently improved blood-gas exchange and respiratory function, finally prolonged the survival time of BLM-induced fibrosis mice. Furthermore, PDX inhibited EMT in a dose-dependent manner in ATII cells. The effect of PDX on fibrosis *in vivo* and *in vitro* is summarized in Fig. 7.

TGF-β is a key mediator in fibrosis which stimulates ECM synthesis^17^. In our data, the level of TGF-β was increased in BLM group and decreased by PDX posttreatment. Recent studies have shown many innate proinflammatory cytokines have crucial roles in the pathogenesis of fibrosis. Mice that overexpressed IL-17, TNF-α and IL-1β in the lung developed highly progressive pulmonary fibrosis. TNF-α and IL-1β, in particular, induced EMT and myofibroblast activation via a TGF-β1-mediated mechanism. IL-17 was a contribution to myofibroblast activation, which likely suppressed collagen degradation pathways^18^,^19^,^20^,^21^,^22^. As our data shown, the level of IL-17, TNF-α and IL-1β were increased in BLM group, whereas PDX posttreatment reduced fibrosis related factors. An interesting study showed that challenged with profibrotic IL-17, reduced the expression of several important genes within the autophagy pathway including Atg14 and beclin-1^23^. It is speculated that the attenuation of IL-17 induced by PDX likely promotes autophagy and improves collagen degradation pathways. Therefore, PDX reverses fibrosis possibly through reduction these cytokines to inhibit myofibroblast activation and promote collagen degradation.

Lung function test is an important tool for evaluation of respiratory disease in clinical. Several studies have confirmed that BLM-induced lung fibrosis resulted in a decrease in IC and Cst, an improvement of eslance with or without resistance strengthening^24^,^25^,^26^,^27^,^28^. In our experiments, we performed a systematic and comprehensive assessment of lung function. Our data demonstrated that BLM induced fibrosis presented with a restrictive pulmonary function pattern. The lung function of 7 days after BLM administration presented impaired with a decrease of IC. Upon the methacholine, Rrs and specific airway Rn were intensified, suggesting airway hyperresponsiveness due to inflammation. G and H were enhanced which reflected lung parenchyma injured and phenotype changed, as well as an increased Ers, reinforced lung has presented fibrosis. On day 21, in the BLM group, Rrs and Rn presented a similar extent increase, which was due to the low-grade but persistent inflammatory trigger during fibrosis. PDX reduced respiratory resistance, suggesting PDX promoted fibrosis resolution partly through attenuated inflammatory trigger. Consistently, we found BLM increased Ers, G and H, decreased IC. In addition, BLM also caused a downshift of PV loop curve with an increased Area and a decrease in Cst, A and K, all these values clearly indicated that the intrinsic stiffness of the lung and chest wall was increased and pointed to an improvement elastic recoil of the lung by fibrosis. Posttreatment with PDX restored the compliance and elastance of lung to normal values. Taken together, these results indicate that BLM induced fibrosis not only involved the peripheral lung tissue compartment but also influence the function of the entire respiratory system, which can be restored by PDX.

Refractory hypoxemia by the damage of lung function is the most important cause of death in pulmonary fibrosis^29^. Our results showed that PaO_2_ and SaO_2_ were reduced in BLM group mice, which provided more robust evidence for the successful establishment of the fibrosis model. However, the PH, PaCO_2_, and Lac had no significant change between the saline group and the BLM group, which may be related to the chronic progress of fibrosis. Mice would still be in the stage of compensatory on day 21 after BLM challenge, and mice would be adapted to the lower oxygen so as to no change in the level of PH, which could be supported by the level of Lac. Consistent with us, previous researches also have shown a similar effect that the PH, PaO_2_ and SaO_2_ were decreased in BLM-induced fibrosis mice, with or without a significantly increase in PaCO_2_^30^,^31^.

The key cellular mediator in fibrosis is the myofibroblast. In mouse models of renal and hepatic fibrosis, up to 40% of α-SMA-positive myofibroblasts have been showed derived from epithelial cells via EMT. TGF-β is a crucial stimulator of the EMT. TGF-β induced EMT for epithelial cells initiate metastasis which leads to switching of E-cadherin to N-cadherin^32^. In our study, we proved that TGF-β decreased E-cadherin protein expression and induced N-cadherin, α-SMA protein expression of ATII cells, whereas PDX inhibited this process *in vivo* and *in vitro*. It is suggesting that PDX exerts a therapeutic effect on fibrosis partly through modulating epithelial phenotypes, regulating the balance of EMT and mesenchymal–epithelial transition (MET).

In conclusion, this study demonstrates that posttreatment with PDX, ameliorated lung fibrosis induced by BLM in mice. In addition, we also demonstrate that PDX improved lung function, enhanced blood-gas exchange, and prolonged the life span. Finally, we find PDX inhibited EMT *in vivo* and *vitro*. These data have significant implications for future efforts in developing an efficient therapeutic strategy for treating lung fibrosis by targeting PDX actions. It is noteworthy that we show the therapeutic effect of PDX, given that in clinical use is often more important when the fibrotic changes of various etiologies are already apparent in the patient. Future experiments are necessary to understand the basic mechanism underlying the therapeutic effects.

## Materials and Methods

### Reagents

PDX (10(S),17(S)-Dihydroxy-docosa-4Z,7Z,11E,13Z,15E,19Z-hexaenoic acid) was obtained from Cayman Chemical Company (Michigan, USA). BLM, methacholine were purchased from Sigma Company (Santa Clara, USA). IL-1β, IL-17, TNF-α, TGF-β ELISA kits were purchased from R&D Systems. Antibodies against anti-alpha smooth muscle actin (α-SMA) antibody and fibronectin were obtained from Abcam Company (Cambridge, UK). Antibodies against E-cadherin and N-cadherin were from Cell Signaling Technology Company (Boston, USA). Recombinant Human TGF-β1 (HEK293 derived) was from Peprotech Company (Rocky Hill, USA).

### Animal Model of Fibrosis

C57BL/6 mice at 6–8 wk of age were purchased from the Shanghai SLAC Laboratory Animal Co. Ltd. Principles of laboratory animal care were followed and all procedures were conducted according to the guidelines established by the National Institutes of Health (NIH Publication No. 85–23, revised 1996), and every effort was made to minimize suffering. This study was approved by the Animal Care and Use Committee of Wenzhou University. The animals were acclimatized for 7 days prior to experimental use. Mice were caged with free access to food and fresh water in a temperature-controlled room (22–24 °C) on a 12-h light/dark cycle. For the induction of pulmonary fibrosis, mice were challenged with BLM (2 mg/kg, 2 mg/ml dissolved in saline) by a noninvasive orotracheal intubation under direct vision with a 24 G vein remaining needle or given equivalently sterile saline as a control. We defined the day of BLM administration as day 0. Mice were anesthetized on day 7 after BLM inoculation for pulmonary function assessment and then the lungs were removed for histological analysis. To evaluate the effect of late treatment, PDX was given i.p. on day 8 at 1 μg/mouse and then boosted at 100 ng/mouse every two days. Mice were anesthetized for arterial blood-gas analysis and lung function test on day 21 after BLM challenge and lungs were removed for fibrosis test.

**Figure 8:**
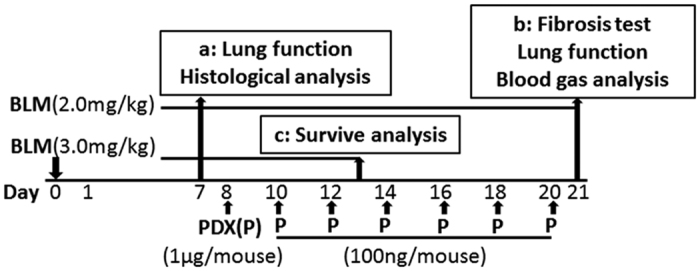
Experimental schedule. Mice were treated with bleomycin (BLM) (or equivoluminal saline as a control) by the intratracheal (i.t.) route at 2.0 mg/kg, we defined the day of bleomycin administration as day 0. Starting on day 8, mice were injected intraperitoneally (i.p.) with Protectin DX (PDX) (1 μg/mouse) followed by boosted 100 ng/mouse every two days. Alcohol is resolvent. Mice were divided into five groups: saline, BLM, BLM + PDX, BLM + Alcohol, and PDX. As part of a time course experiments, on days 7 (**a**) and 21 (**b**), mice were prepared for lung fibrosis analysis. (**c**) For survive experiments, mice were challenged with BLM at 3.0 mg/kg followed by posttreatment with PDX.

For survival experiment, mice were administrated BLM (3.0 mg/kg) by intratracheal and treated with PDX from day 8. At the same time, mice weight in three groups was recorded every day. The experimental schedule is shown in Fig. 8.

### Histological Analysis

For histological examination, on days 7 and 21 after BLM administration, mice were killed and the left lung tissues were fixed with 10% paraformaldehyde for 24 hours, embedded in paraffin wax sectioned (4 μm thicknesses). The pulmonary tissue slides were stained with H&E, Masson for light microscope analysis. Ashcroft fibrosis score was used for grading fibrosis scale.

### Transformation electron microscope

Blocks were rinsed overnight in 0.1 M phosphate buffer (350 mOsm, pH 7.4) and fixed for two hours in osmium tetroxide (1% osmium tetroxide in 0.125 sodium cacodylate buffer, 400 mOsm, pH 7.4). The samples were then passed through stepwise dehydration in increasing concentrations of ethanol (50–100%), rinsed with propylene oxide and embedded in Araldite. Blocks were then cut into ultra thin sections (50–70 nm) and contrast stained with saturated uranyl acetate and bismuth subnitrate. Sections were examined at an accelerating voltage of 60 kV using a Zeiss EM 10 C transformation electron microscope(HITACHIH-7500). Micrographs of a carbon grating replica were taken for calibration.

### Immunohistochemistry

After paraffin removal in xylene, the sections were rehydrated and were placed in a pressure cooker with citrate buffer, pH 6.0, for 1 min after reaching boiling temperature to retrieve antigenic sites masked by formalin fixation. After quenching of endogenous peroxidase with 3% of H_2_O_2_ for 15 min, sections were incubated with antibody to α-SMA, fibronectin, E-cadherin, N-cadherin, (1:100 dilution) or with the preimmune serum as a negative control stain overnight at 4 °C, and subsequently were incubated for 1 h at room temperature with the biotinylated anti-rabbit IgG and peroxidase-conjugated streptavidin, with diaminobenzidine. Slides were counterstained for 30 seconds with hematoxylin. Observed and photographed with microscopy (Nikon eclipse 90i, Tokyo, Japan).

### ELISA

Right lung tissue samples were homogenized in 50 mM potassium phosphate buffer (PB, pH 6.0). After three freeze and thaw cycles, the samples were centrifuged at 12,000 rpm for 20 min at 4 °C, then subpackaged and stored at −80 °C. IL-1β, IL-17, TNF-α, and TGF-β1 were measured by ELISA kits. All procedures were done in accordance with the manufacturer’s instructions.

### Hydroxyproline assay

Lung hydroxyproline content was determined by an acid hydrolysis assay according to the manufacturer’s instructions (Nanjing Jiancheng Bioengineering Institute, China).

### Arterial blood gas analysis

Blood samples obtained from the abdominal aorta were assessed using the ABL90 FLEX blood-gas analyzer (Radiometer, Copenhagen, Denmark) at room temperature. Arterial oxygen partial pressure (PaO_2_), arterial carbon dioxide partial pressure (PaCO_2_), arterial oxygen saturation (SaO_2_), pH, and lactic acid (Lac) concentrations of the samples were recorded.

### Invasive assessment of respiratory mechanics

Mice were anesthetized on days 7 and 21 after BLM administration with 70–90 mg/kg pentobarbital sodium and tracheotomized. To cause diaphragm paralysis, Vecuronium Bromide was injected via tail intravenous and then mechanically ventilated at a rate of 150 breaths/min, tidal volume of 12 ml/kg, and a positive end-expiratory pressure (PEEP) of 3 cm H_2_O with a computer-controlled small-animal ventilator. Airway pressure, volume, and airflow were recorded using a controlled piston to evaluate lung mechanics.

Measurements of the respiratory system mechanics have been assessed using the flexiVent system (Scireq, Montreal, QC, Canada) and evaluated assuming four different models. Deep Inflation is a pressure-driven perturbation, to calculate the resulting changes in volume under a controlled pressure. Pressure-volume (PV) curve assesses the distensibility of the respiratory system over the entire inspiratory capacity. SnapShot-150 is a single compartment model, reflecting overall lung resistance, elastance and compliance. Quick Prime-3(QP-3) is a constant phase model, using a forced oscillation to separate central and peripheral airways.

Inspiratory capacity (IC) was recorded by a Deep Inflation perturbation. PV curves were generated by a sequential and increasing delivery of air into the lungs from resting pressure (zero volume) to total lung capacity followed by sequential expiratory steps during which air was incrementally released. A (estimate of inspiratory capacity), K (shape parameter), Cst (quasi-static compliance) and Area (hysteresis, area in PV loop) were determined from the analysis of PV curves. Total respiratory system resistance (Rrs), and elastance (Ers) were measured by the SnapShot-150. Newtonian resistance (Rn), a close approximation of resistance in the central airways, G (tissue damping) reflecting energy dissipation in the lung tissue, and H (tissue elastance) reflecting energy storage in the tissue were obtained from QP-3.

After performing all perturbations at a baseline level, mice were challenged with increasing methacholine aerosols, generated with an in-line nebulizer (5 s) and administered directly with increasing concentrations (0 = saline, 3, 9 and 27 mg/ml). To measure Rrs and Ers, the Snapshot-150 was performed. With a forced oscillation manoeuvre, Rn, G and H were recorded. Changes in reactivity and sensitivity were assessed using non-linear regression analysis to calculate the responses to each methacholine dose. The maximum response of each methacholine dose for above parameters was assessed. All data was analyzed using flexiVent software (version7.6).

### Western Blot

Western blot analysis from frozen lungs and cells homogenates were performed as described previously. After equal amounts of protein were loaded in each lane and separated by 10% SDS-PAGE, the proteins were transferred to polyvinylidene difluoride membranes. The membranes were blocked for 2 h with 5% skimmed milk, which was also used as primary and secondary antibodies incubation buffer. The primary antibodies were used at dilutions of 1:1,000, and incubated overnight at 4°C. Horseradish peroxidase conjugated secondary antibodies, which were either goat anti-mouse or goat anti-rabbit, were used at 1:3,000 dilution and imaged with the Image Quant LAS 4000 mini (GE Healthcare Bio-Sciences AB, Uppsala, Sweden).

### Primary rats ATII cells isolation, culture, and treatment

Primary rats alveolar type II epithelial (ATII) cells were isolated from Sprague–Dawley rats (200–250 g) from the Shanghai SLAC Laboratory Animal Co. Ltd by elastase digestion of lung tissue and then differentially adhered on IgG-coated plates as described by Dobbs *et al*.^33^ ATII cells were seeded onto plastic culture dishes at 2–4 × 10^6^/cm^2^ and cultured in a 5% CO_2_, 95% air atmosphere in DMEM containing 10% FBS, 2 mM L-glutamine, 100 U/ml penicillin, and 0.1 mg/ml streptomycin after isolation. For all experiments, cells were cultured into six-well plates and maintained until subconfluence (80%), and cells were serum deprived for 24 h before the addition of TGF-β (10 ng/ml) in the presence or absence of PDX.

### Statistical Analysis

All values were reported as mean ± SEM. Data were analyzed by Student’s *t* test for two-group comparison and one-way ANOVA followed by Tukey’s post-hoc test for multiple comparisons. Mechanical data were evaluated using Two-way ANOVA followed by multiple comparisons test. Kaplan–Meier survival analysis was used and statistical significance was determined by log-rank (Mantel-Cox) test. Analysis and graphs were done with GraphPad Prism 6.0 (GraphPad, San Diego, CA, USA). Results with P < 0.05 were considered statistically significant.

## Additional Information

**How to cite this article:** Li, H. *et al*. Posttreatment with Protectin DX ameliorates bleomycin-induced pulmonary fibrosis and lung dysfunction in mice. *Sci. Rep.* **7**, 46754; doi: 10.1038/srep46754 (2017).

**Publisher's note:** Springer Nature remains neutral with regard to jurisdictional claims in published maps and institutional affiliations.

## Supplementary Material

Supplementary Information
