# Supplementary material for: Posttreatment with Protectin DX ameliorates bleomycin-induced pulmonary fibrosis and lung dysfunction in mice
Source: Sci Rep. 2017 May 3;7:46754. doi: 10.1038/srep46754 (PMC5413938; doi:10.1038/srep46754)
Supplement: Supplementary Information [file srep46754-s1.pdf]

Posttreatment with Protectin DX ameliorates bleomycin-induced pulmonary fibrosis and lung dysfunction in mice

Hui Li[1], Yu Hao[1], Huawei Zhang[1], Weiyang Ying[1], Dan Li[1], Yahe Ge[1], Binyu Ying[1], Bihuan Cheng[1], Qingquan Lian[1 †] and Shengwei Jin[1 †]

[1]Department of Anesthesia and Critical Care, Second Affiliated Hospital of Wenzhou Medical University, Zhejiang 325027, China

[†] Corresponding Author:

Shengwei Jin, MD, PhD E-mail: jinshengwei69@163.com

Qingquan Lian, MD, PhD E-mail: lianqingquanmz@163.com

Address: Department of Anesthesia and Critical Care, Second Affiliated Hospital and Yuying Children's Hospital of Wenzhou Medical University, 109 Xueyuan Road, Wenzhou, Zhejiang Province, P. R. China 325027.

Telephone: 0086-577-88002806 Fax number: 0577-88832693

Supplementary Figures

Figure S1

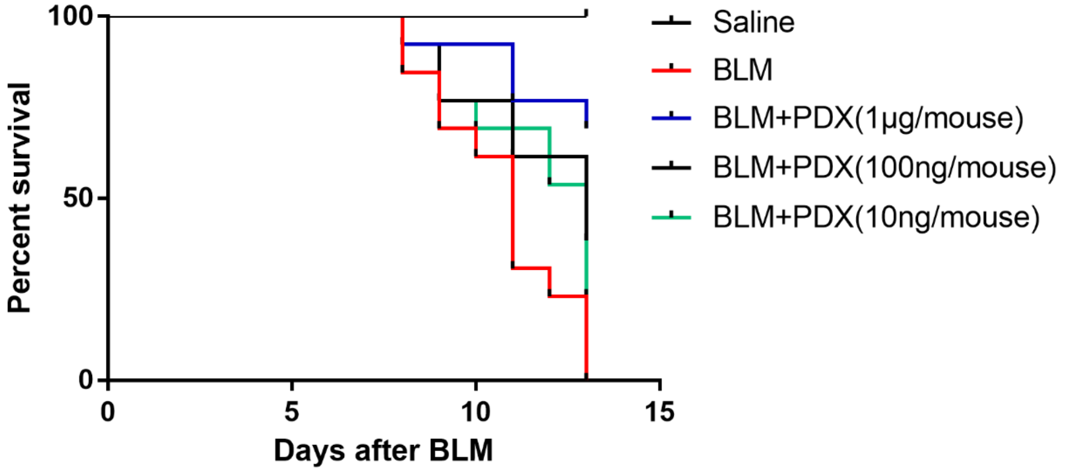

Figure S1. The effect of different doses of PDX on bleomycin(BLM) induced fibrosis mice. there were five groups: saline group, BLM group, BLM plus PDX (1μg /mouse) group, BLM plus PDX (100ng/mouse) group; BLM plus PDX (10ng/mouse) group. After BLM (3.0mg/kg) inuistillation, the three BLM plus PDX groups received intraperitoneal injection of PDX (1μg/mouse, 100ng/mouse, 10ng/mouse respectively) on day 8 after BLM administration and boost 1/10 every other day. Control group received saline only. The survival rates of five groups of mice were shown over a 13-day observation period. Data was expressed as percentage of mice alive at each time point. n=13 for each group.

Figure S2

A B

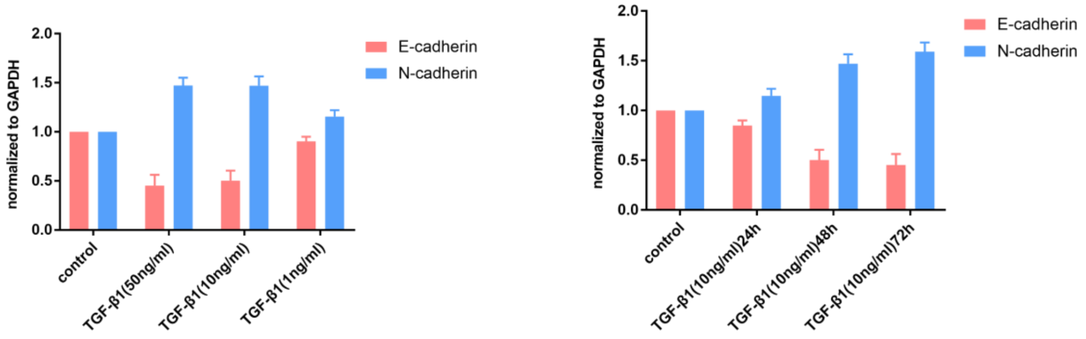

Figure S2. The effect of TGF-β1 in different doses and time points on primary rats alveolar type II epithelial (ATII) cells. ATII cells were cultured into six-well plates and were serum deprived for 24 h before the addition of TGF-β1. The effect of TGF-β1 on ATII cells at concentrations of 50ng/ml, 10 ng/ml, 1 ng/ml respectively (A). The effect of TGF-β1 on ATII cells for 24h, 48h, 72h respectively (B). The expression of E-cadherin and N-cadherin were detected by real-time quantitative RT-PCR. Data were presented as means ±SEM. n=5 for each experimental group. (methods details see the *Supplementary Methods*)

Supplementary Tables

Table S1. Real-Time RT-PCR Primer Sequences

Rat Genes	Forward Primers	Reverse Primers
E-cadherin	CCTAAGCACAACAGCAAAAGC	AATACGGGCACCGACCTCA
N-cadherin	AAGGCAATCCCACTTACGG	TTGGCTACAATGACATCCACTC
GAPDH	GGCTCTCTGCTCCTCCC	CCGTTCACACCGACCTT

Supplementary Methods

Real-time PCR

Total RNA was extracted using the Trizol reagent (Invitrogen), according to the manufacturer’s instructions. E-cadherin and N-cadherin mRNA were quantified by real-time PCR assay using the SYBR Green real-time PCR Master Mix regents (Toyobo, Osaka, Japan) with glyceraldehyde-3-phosphatedehydrogenase (GAPDH) as the normalization control. Real-time PCR reactions were done on the CFX96 Real-Time PCR Detection System (Bio-Rad, Hercules, CA, USA).
